# Supplementary material for: Mdm2-Mediated Downmodulation of GRK2 Restricts Centrosome Separation for Proper Chromosome Congression
Source: Cells. 2021 Mar 25;10(4):729. doi: 10.3390/cells10040729 (PMC8064503; doi:10.3390/cells10040729)
Supplement: Supplementary file 1 [file cells-10-00729-s001.pdf]

Mdm2-mediated downmodulation of GRK2 restricts centrosome separation for proper chromosome congression

Clara Reglero [1,4,5], Belén Ortiz del Castillo[1,2,5], Verónica Rivas[1,2], Federico Mayor jr[1,2,3] and Petronila Penela*[1,2,3,6]

SUPPLEMENTARY FIGURE LEGENDS

Fig. S6. Scheme of the Mdm2 role in the control of GRK2 for proper centrosome separation and length of the mitotic spindle. Phosphorylation of GRK2 at Ser670 promotes Pin1 binding and Mdm2-mediated ubiquitination, which eventually leads to GRK2 proteasome degradation. In addition, phosphorylation of S670 catalytically activates GRK2 towards Mst2 which in turn stimulates Nek2A to initiate centrosome separation. Hence, the protein modification of GRK2 enabling activity of the GRK2/Mst2/Nek2A axis for separation also switches-on Mdm2 degradation of GRK2, and these two processes must be properly balanced for precise distancing of centrosomes during G2 and spindle length in mitosis. Thus, defective Mdm2 activity causes stabilization of S670-phophorylated GRK2 protein which in turn activate the Mst2/Nek2A axis, leading to increased centrosome separation in G2 phase. Conversely, weak activation of Mst2/Nek2A and lower distances between centrosomes could result from excessive Mdm2-mediated degradation of GRK2. Similar effect is promoted with overexpression of the GRK2-S670A mutant that shown impaired GRK2 down-modulation but fails to activate Mst2, also resulting in spindle shortening in mitosis, while overexpression of the wild-type GRK2 protein triggers the opposite effect.

SUPPLEMENTARY FIGURES (S1-S6)

Figure 1A

Low exposure

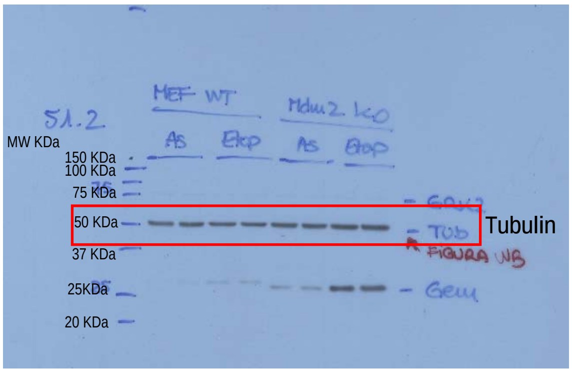

high exposure

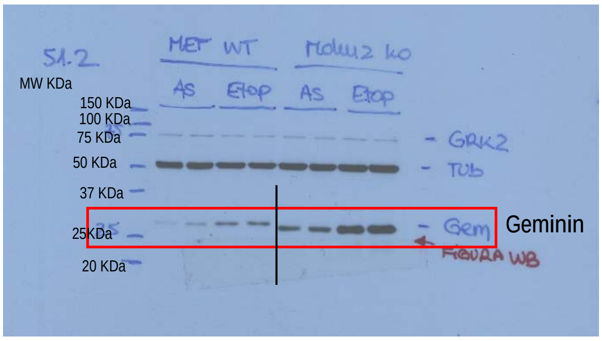

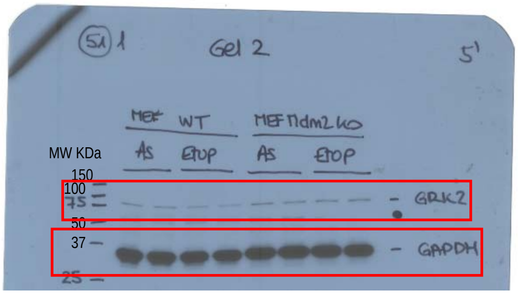

Figure 1B

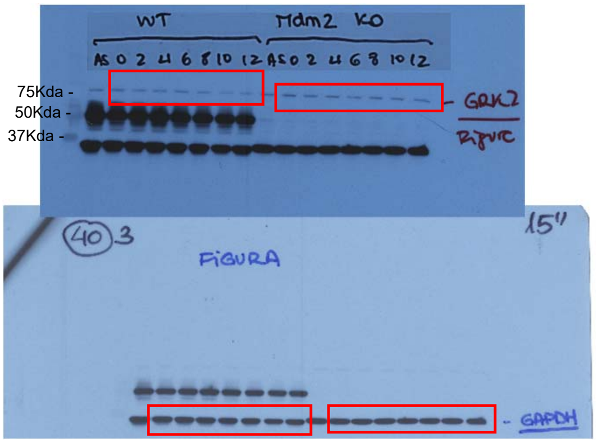

Figure 1B

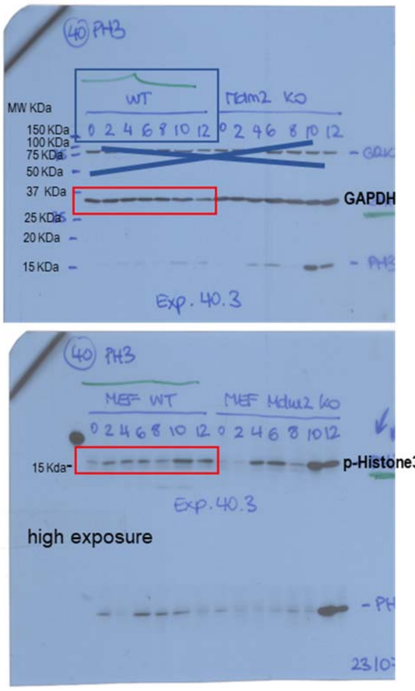

Figure 1B

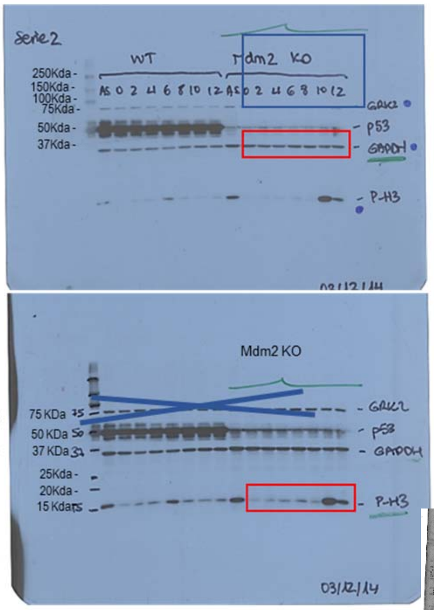

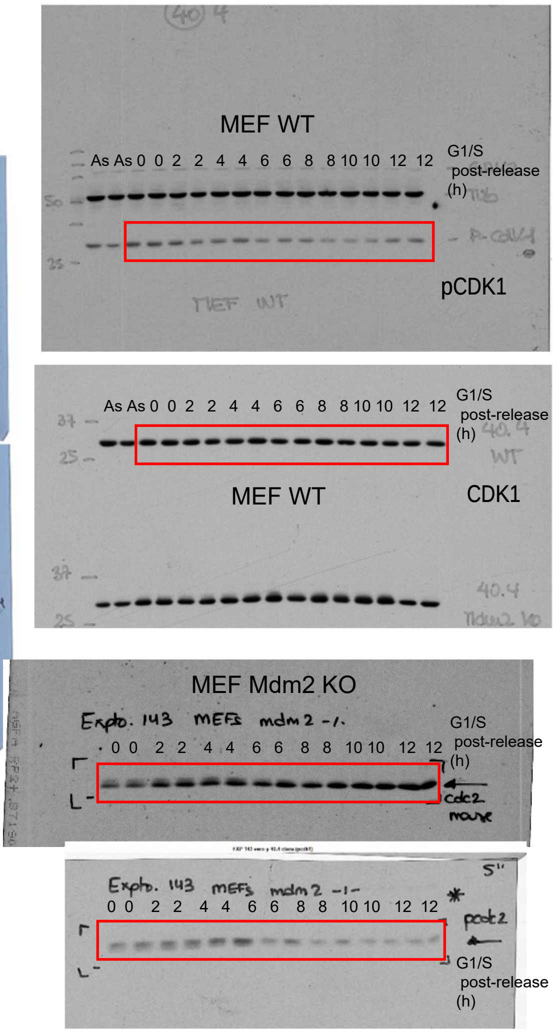

Figure S1. Detailed information about western blot in Figure 1.

Figure 2A

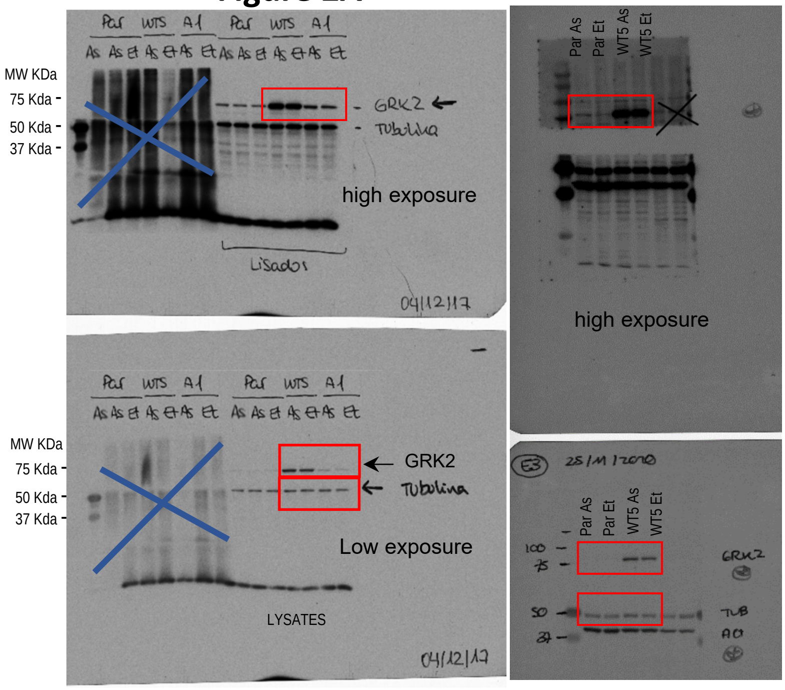

Figure 2B

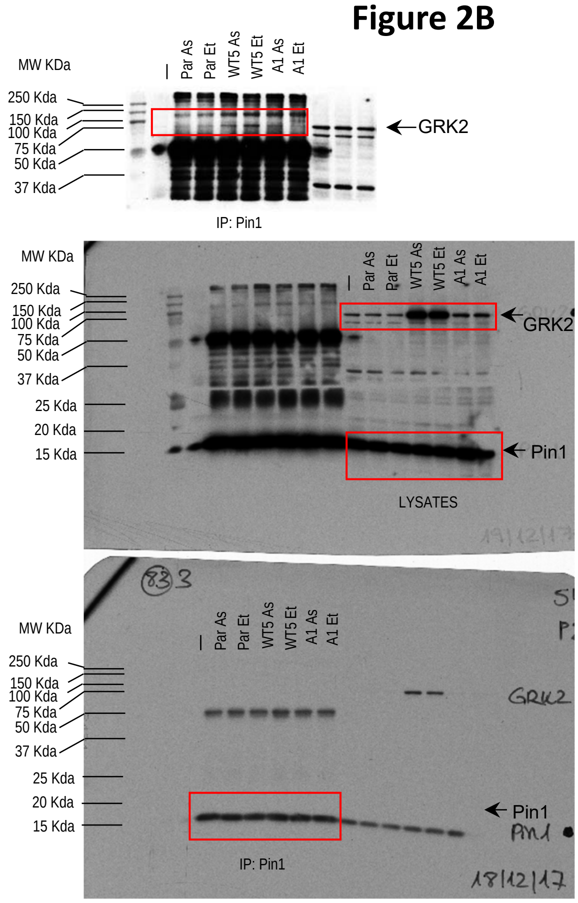

Figure 2D

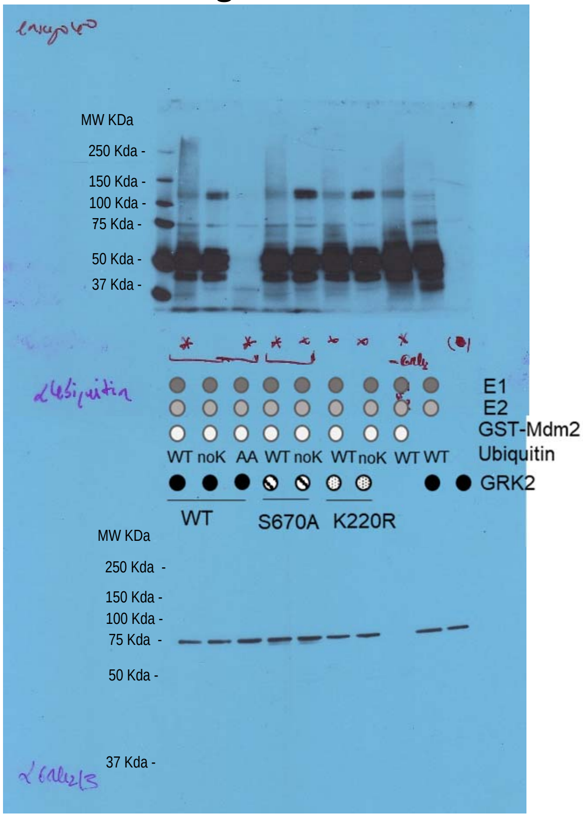

Figure 2C

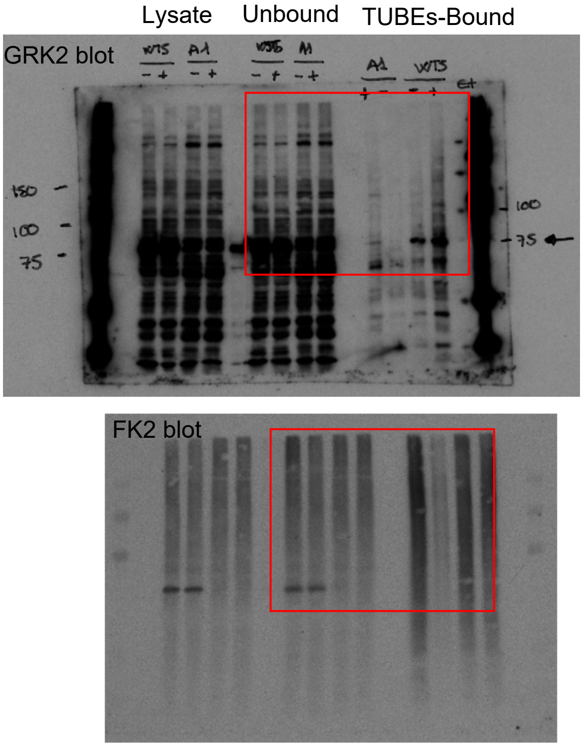

Figure 2E

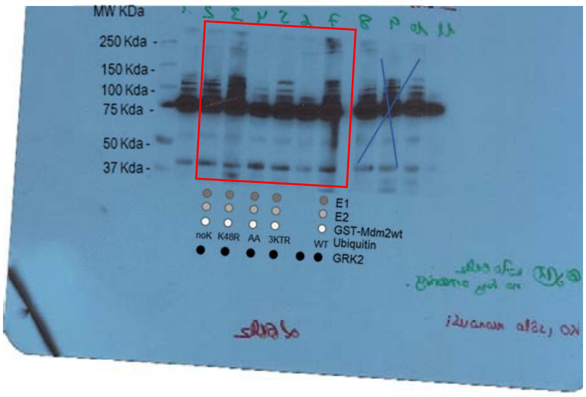

Figure S2. Detailed information about western blot in Figure 2.

Figure 3A

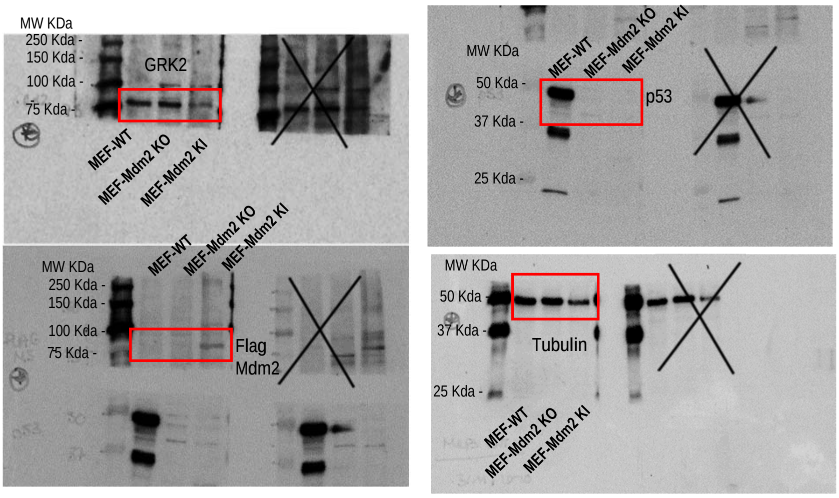

Figure 3C

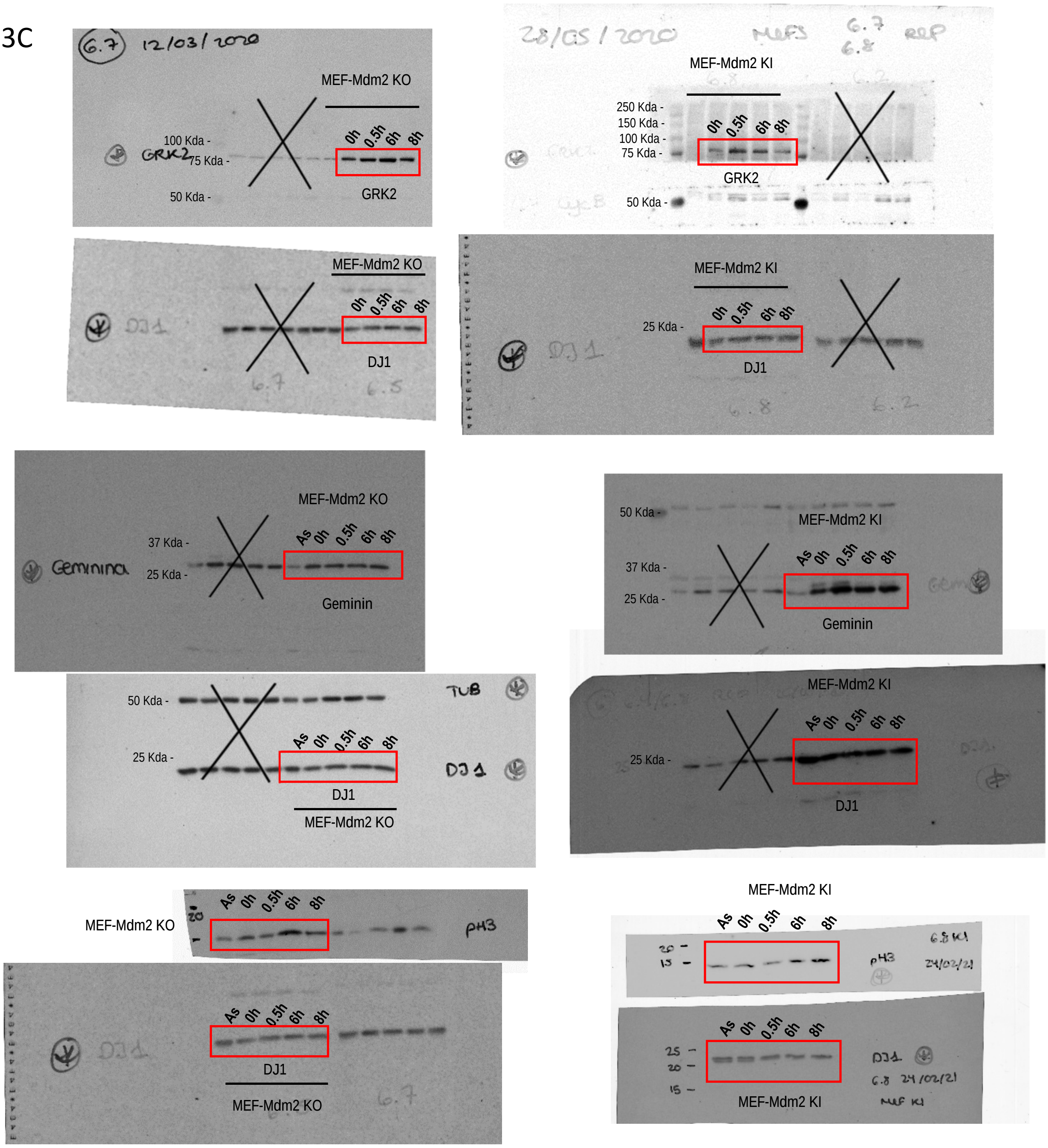

Figure S3. Detailed information about western blot in Figure 3.

Figure 4D

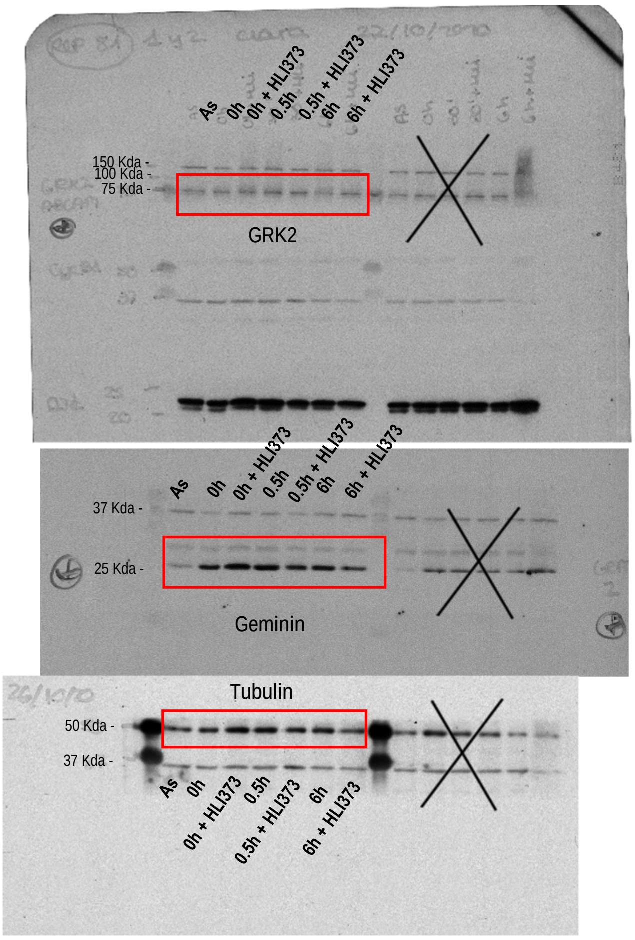

Figure S4. Detailed information about western blot in Figure 4.

Figure 5A

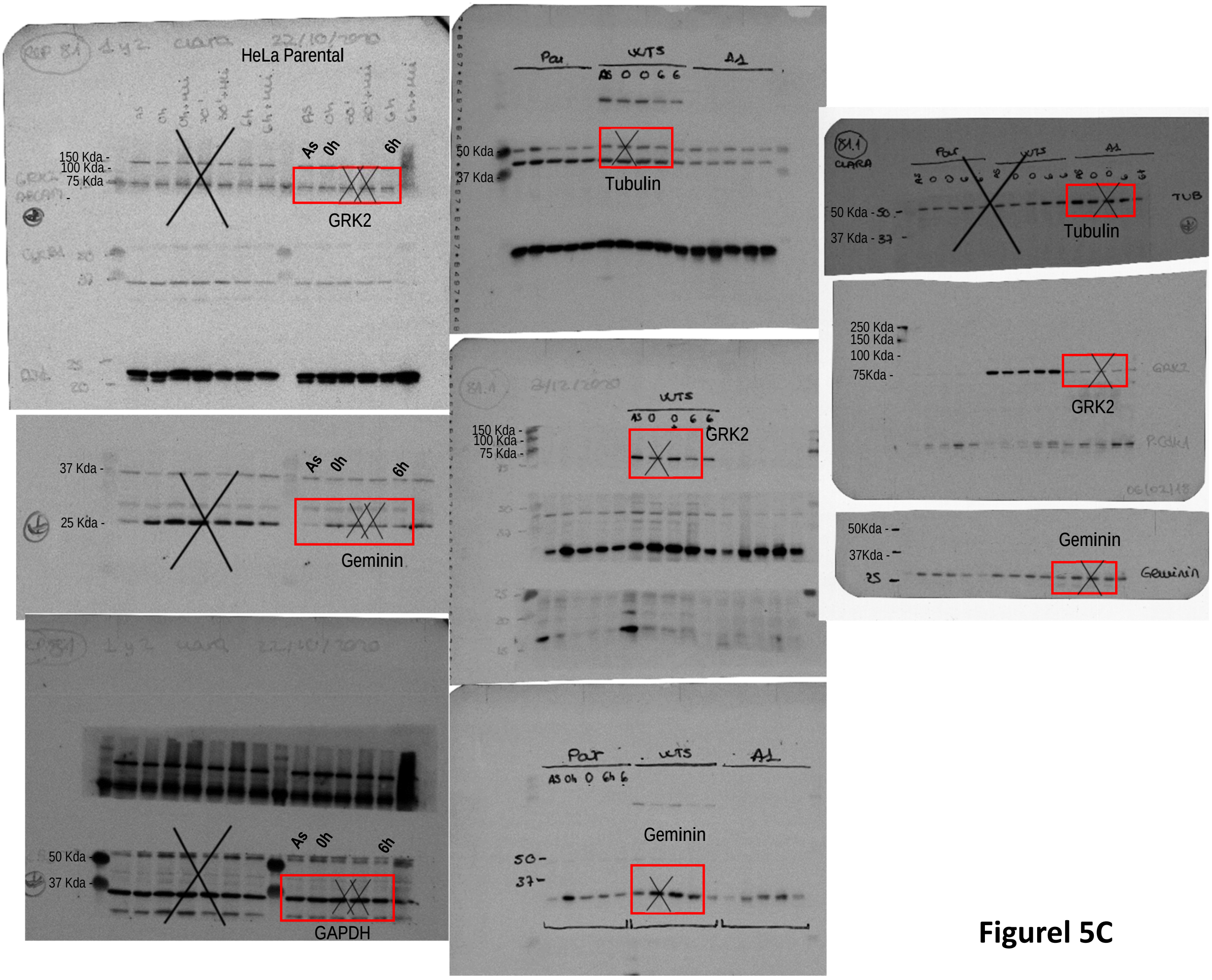

Figurel 5C

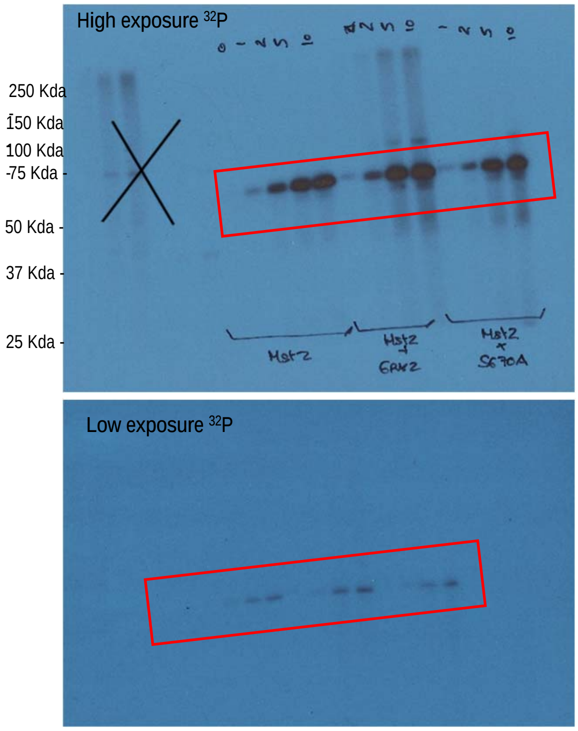

Figurel 5C

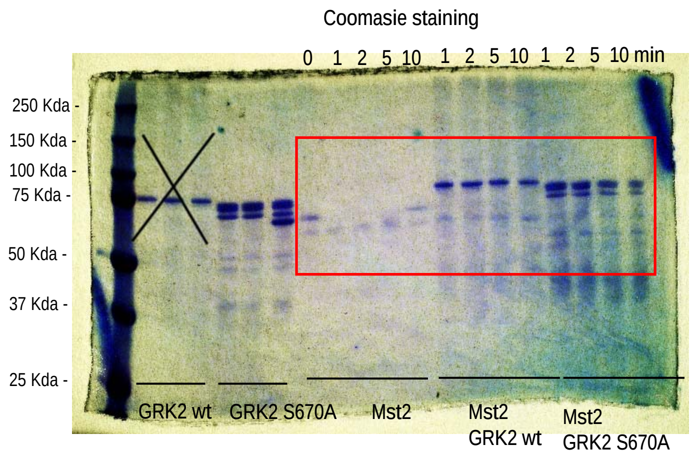

Figure S5. Detailed information about western blot in Figure 5.

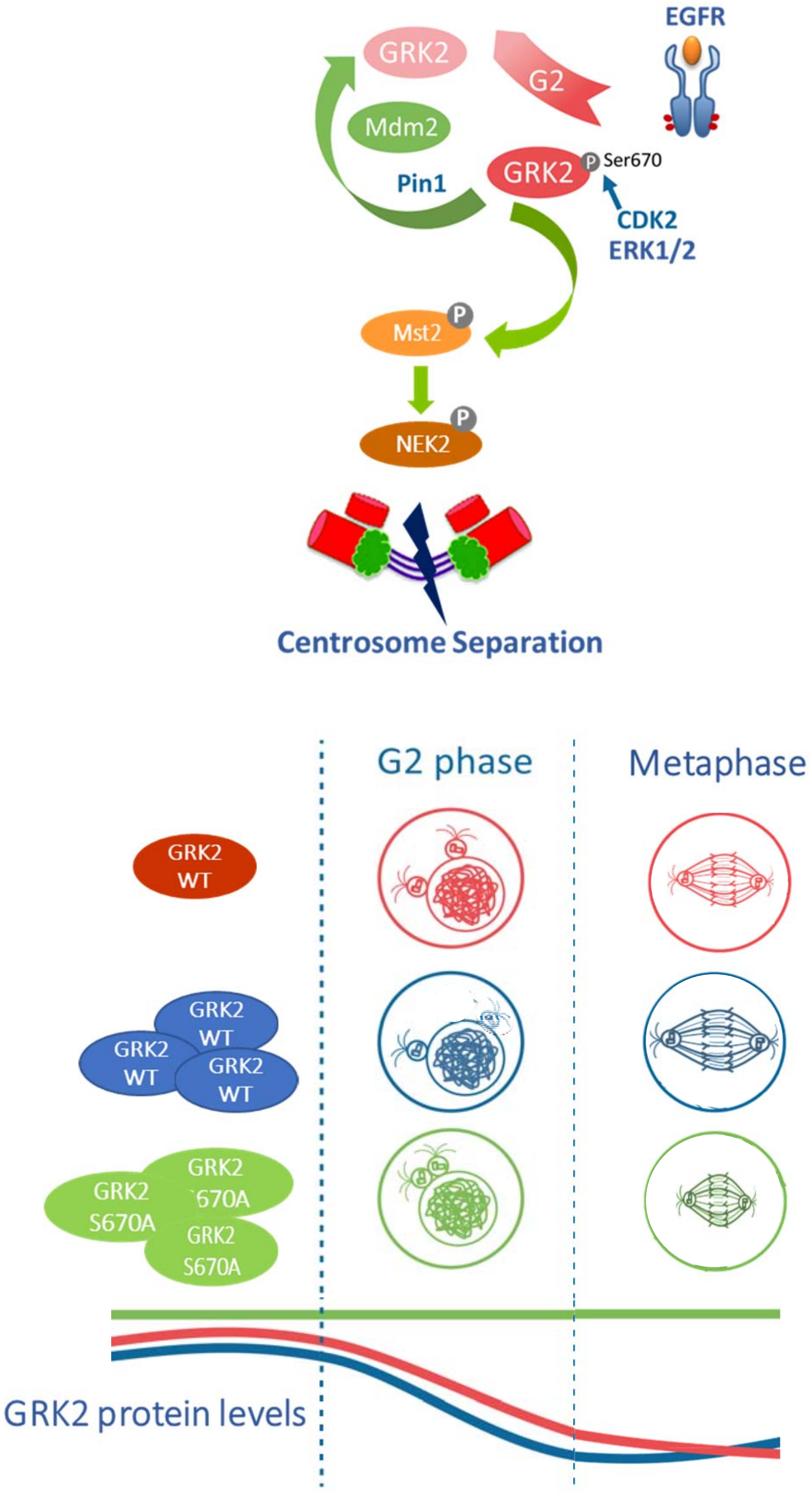
EGFR
GRK2
G2
Mdm2
Pin1
GRK2
P
Ser670
CDK2
ERK1/2
Mst2
P
NEK2
P
Centrosome Separation
G2 phase
Metaphase
GRK2
WT
GRK2
WT
GRK2
WT
GRK2
WT
GRK2
S670A
GRK2
S670A
GRK2
S670A
GRK2 protein levels

Figure S6
